# Hedgehog signaling in humans: the HH_Signal pathway_db knowledge base

**DOI:** 10.18699/vjgb-25-103

**Published:** 2025-12

**Authors:** T.A. Bukharina, A.M. Bondarenko, D.P. Furman

**Affiliations:** Institute of Cytology and Genetics of the Siberian Branch of the Russian Academy of Sciences, Novosibirsk, Russia; Novosibirsk State University, Novosibirsk, Russia; Institute of Cytology and Genetics of the Siberian Branch of the Russian Academy of Sciences, Novosibirsk, Russia Novosibirsk State University, Novosibirsk, Russia

**Keywords:** knowledge base, Hedgehog signaling pathway, morphogenesis, evolution, gene networks, regulatory circuits, база знаний, сигнальный путь Hedgehog, морфогенез, эволюция, генные сети, регуляторные контуры

## Abstract

The rapid advancement of omics technologies (genomics, transcriptomics, proteomics, metabolomics) and other high-throughput methods for experimental studies of molecular genetic systems and processes has led to the generation of an unprecedentedly vast amount of heterogeneous and complex biological data. Effective use of this information resource requires systematic approaches to its analysis. One such approach involves the creation of domain-specific knowledge/data repositories that integrate information from multiple sources. This not only enables the storage and structuring of heterogeneous data distributed across various resources but also facilitates the acquisition of new insights into biological systems and processes. A systematic approach is also critical to solving the fundamental problem of biology – clarifying the regularities of morphogenesis. Morphogenesis is regulated through evolutionarily conserved signaling pathways (Hedgehog, Wnt, Notch, etc.). The Hedgehog (HH) pathway plays a key role in this process, as it begins functioning earlier than others in ontogenesis and determines the progression of every stage of an organism’s life cycle: from structuring embryonic primordia, histo- and organogenesis, to maintaining tissue homeostasis and regeneration in adults. Our work presents HH_Signal_pathway_db, a knowledge base that integrates curated data on the molecular components and functional roles of the human Hedgehog (HH) signaling pathway. The first release of the database (available upon request at bukharina@bionet.nsc.ru) contains information on 56 genes, their protein products, the regulatory interaction network, and established associations with pathological conditions in humans. HH_Signal_pathway_db provides researchers with a tool for gaining new knowledge about the role of the Hedgehog pathway in health and disease, and its potential applications in developmental biology and translational medicine.

## Abstract (ru)

Стремительное развитие омиксных технологий (геномики, транскриптомики, протеомики, метаболомики) и других высокопроизводительных методов экспериментального исследования молекулярно-генетических систем и процессов привело к генерации беспрецедентно огромных объемов разнородных и сложных биологических данных. Эффективное использование этого информационного ресурса требует системных подходов к их анализу. Один из подходов состоит в создании предметно-ориентированных баз знаний/данных – репозиториев, интегрирующих информацию из множества источников, что позволяет не только хранить и структурировать распределенные по различным источникам гетерогенные данные, но и получать новые сведения о биологических системах и процессах. Критически важен системный подход и к решению фундаментальной задачи биологии – выяснению закономерностей морфогенеза. Регуляция морфогенеза осуществляется через эволюционно консервативные сигнальные пути (Hedgehog, Wnt, Notch и др.). Ключевая роль в этом процессе принадлежит пути Hedgehog (HH), поскольку в онтогенезе он начинает функционировать ранее других и детерминирует реализацию каждого этапа индивидуального развития организма: от структурирования эмбриональных зачатков, гисто- и органогенеза до поддержания тканевого гомеостаза и процесса регенерации у взрослых особей. Нами создана база знаний HH_Signal_pathway_db, в которую сведена информация о компонентах и функциях HH сигнального пути у человека. Первый релиз базы (доступен по запросу bukharina@bionet.nsc.ru) содержит информацию о входящих в него 56 генах, их белковых продуктах, сети регуляторных взаимодействий, а также об установленных связях с некоторыми патологическими состояниями человека. HH_Signal_pathway_db предоставляет исследователям инструмент для получения новых знаний о роли пути Hedgehog в норме и при патологии и возможностях применения их в области биологии развития и трансляционной медицины.

## Introduction

Modern molecular-genetic and biomedical studies using advanced techniques generate vast amounts of heterogeneous information (Regev et al., 2017; Schermelleh et al., 2019, Kenneth, 2022). This includes data obtained during investigations of various aspects of morphogenesis – a fundamental process leading to the formation of intricate organism architecture. Understanding the mechanisms underlying morphogenesis is essential not only for answering one of biology’s most profound questions – how a single cell gives rise to a highly complex, spatially organized multicellular organism – but also for explaining the mechanisms of tissue regeneration, the causes of congenital anomalies, and pathological conditions of various etiologies, including oncological diseases

Numerous genes, proteins, miRNAs, and signaling molecules are involved in regulating morphogenesis (ENCODE Project Consortium, 2012; Briscoe, Thérond, 2013; Bartel, 2018; Ghafouri-Fard et al., 2022; McIntyre et al., 2024). Some of these components belong to specific signaling pathways.

Signaling pathways (signal transduction) act as transmitter of signals received at the external cell membrane into the nucleus. Cascades of intermolecular interactions involving ligands, receptors recognizing those ligands, intracellular signal transducers of both protein and non-protein nature, transcription factors and co-regulators, etc., mediate pathways. The outcome of pathways’ activity is alteration of target gene expression and corresponding protein levels, which ultimately leads to changes in the functional state of the cell.

Signaling pathways in animals and humans are evolutionarily conserved, and their roles are similar across different taxonomic groups. The pathways constitute complex networks characterized by crosstalk, and the development of a fullyfunctional organism requires the precise coordination of their activities. Signaling pathways are critically important for normal ontogenesis, mutations or alterations in gene expression within these pathways can lead to severe developmental disorders (Artavanis-Tsakonas et al., 1999; Ingham, McMahon, 2001; Logan, Nusse, 2004; Rubin, 2007; Perrimon et al., 2012; Briscoe, Thérond, 2013; Huttlin et al., 2017).

The Hedgehog (HH) signaling pathway, which owes its name to the discovery of the hedgehog (hh) gene in Drosophila melanogaster in the early 1980s, plays a substantial role in controlling morphogenesis. The larvae of flies mutant for this gene are covered with spines, giving them a hedgehog-like appearance (Nüsslein-Volhard, Wieschaus, 1980).

**Fig. 1.**
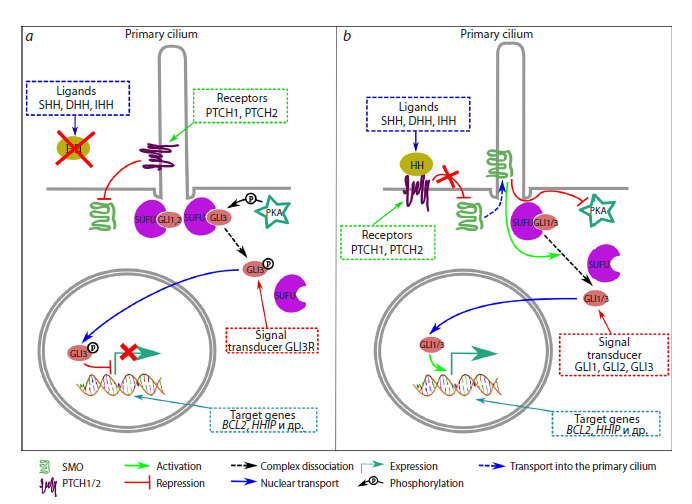
General scheme of the human Hedgehog signaling pathway. a – the mechanism of action when no HH ligand is present; b – the mechanism when PTCH1/2 receptors bind to HH ligands (details explained in text).

The Hedgehog signaling pathway is not merely one of the pathways orchestrating organismal development, but a central regulator of morphogenesis. It determines the anterior-posterior and dorso-ventral body axes and segmentation of embryonic primordia in animals, histo- and organogenesis, and the maintenance of stem cell pools in adult tissues, among other processes. Dysfunction of this signaling pathway is associated with numerous congenital anomalies and human diseases, including cancer of various organs (Ingham, McMahon, 2001; Spinella-Jaegle et al., 2001; Varjosalo, Taipale, 2007; Briscoe, Thérond, 2013; Wu et al., 2017; Skoda et al., 2018; Jamieson et al., 2020; Fitzsimons et al., 2022; Ingham, 2022; Dutta et al., 2023; Jing et al., 2023). It is exactly the reason, that there continues to be unrelenting interest in comprehensive investigation of the molecular-genetic organization and functioning mechanisms of the HH pathway. The general scheme of the Hedgehog signaling pathway is shown in Figure 1.

For the transmission of the HH signal, the recipient cell must contain a specific set of core proteins involved in the process, which must be in certain functional states. These proteins include: the transmembrane receptors Patched1 and Patched2 (PTCH1/2), the inactive form of the transmembrane protein Smoothened (SMO), complexes formed by transcription factors GLI1/3 and scaffold protein Suppressor of fused homolog (SUFU), active protein kinase A (PKA), which is responsible for generating the repressive form of the transcription factor GLI3 (GLI3R).

When the signaling pathway is inactive due to absence of HH ligands (Fig. 1a), PTCH1/2 receptors are localized on the primary cilium – a specialized external organelle of the cell that acts as a sensor for outside signals (Ingham, McMahon, 2001; Eggenschwiler, Anderson, 2007; Oro, 2007; Carballo et al., 2018).

PTCH1/2 block the migration of the SMO protein, which is located in the intracellular space, to the ciliary membrane, and SMO cannot interact with protein kinase A (PKA) to inhibit its activity. As a result, PKA phosphorylates the GLI3/ SUFU complex, the complex dissociates, and GLI3 undergoes proteolytic cleavage to form the repressor GLI3R, which then enters the nucleus and suppresses the transcription of its target genes, including some genes of the HH pathway itself (Gorojankina, 2016; Dilower et al, 2023).

Signal transduction activation occurs when extracellular ligands – proteins belonging to the Hedgehog family (three types exist in humans: Sonic Hedgehog (SHH), Indian Hedge hog (IHH), and Desert Hedgehog (DHH)) – bind to PTCH1/2. The ligand/receptor complex is then removed from the ciliary membrane and transported to the intracellular space, where it is degraded in the lysosome. The position of PTCH1/2 is taken by SMO, which suppresses the activity of protein kinase A, thereby preventing the phosphorylation of the SUFU/GLI3 complex and the formation of GLI3R. Subsequently, within the cilium, the SUFU/GLI1/3 complexes are degraded, and the active forms of GLI1/3 are generated. These enter the nucleus and activate the transcription of target genes, ensuring signal transmission (Ingham, McMahon, 2001; Varjosalo, Taipale, 2007; Briscoe, Therond, 2013; Gorojankina, 2016) (Fig. 1b).

There are two variants of the HH pathway – the canonical one, shown in Figure 1, and the non-canonical one, in which the activation of the GLI1/3 transcription factors occurs without the involvement of SMO, thereby altering the signal transduction route (Brennan et al., 2012; Briscoe, Thérond, 2013; Carballo et al., 2018).

Currently, information concerning the HH pathway in humans is scattered across a vast number of sources (at the time of writing, on request “Hedgehog signaling” in PubMed alone returns 15,247 publications: https://pubmed.ncbi.nlm. nih.gov/?term=hedgehog+signaling), and this body of literature is continually expanding. Despite the extensive growth in the number of studies in this field, a complete and thorough understanding of the evolution, structure, and mechanisms of the HH pathway has not yet been achieved (Ingham et al., 2011; Briscoe; Thérond, 2013; Breeze, 2022).

To integrate, structure, and analyze existing data, the authors are creating a specialized knowledge base HH_Signal_pathways_ db. The database is curated with diverse information related to all aspects of the organization and functioning of the Hedgehog pathway, which enables a systematic approach to its study.

Bioinformatic analysis of the structural and functional organization of the HH pathway opens up opportunities for deeper insight into the molecular-genetic basis of morphogenesis, mechanisms of organ and tissue regeneration, the aging process, the emergence of pathologies of various etiologies, as well as for developing methods for their diagnosis and pharmacotherapy

As part of this work, new results have been obtained, including reconstruction of the associative gene network of the HH signaling pathway, identification of regulatory circuits, and acquisition of data regarding the evolution of genes involved in the pathway.

## Materials and methods

**Fig. 2.**
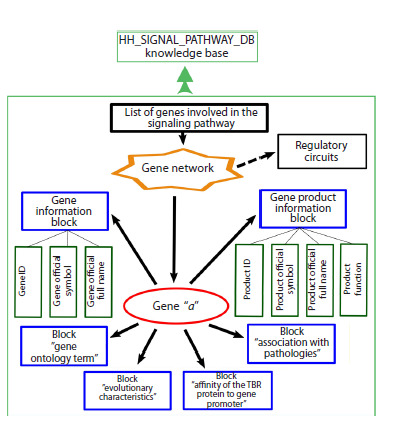
Block diagram of the HH_Signal pathway_db knowledge base.

**Structure and content of the HH_Signal_pathway_db knowledge base**Figure 2 shows a block diagram of the database format developed by the authors.

The list of genes included in the human HH pathway (Table 1) was extracted from the KEGG database (https:// www.genome.jp/kegg/) by querying (Environmental Information Processing→Signal Transduction→Hedgehog Signaling Pathway).

**Table 1.**
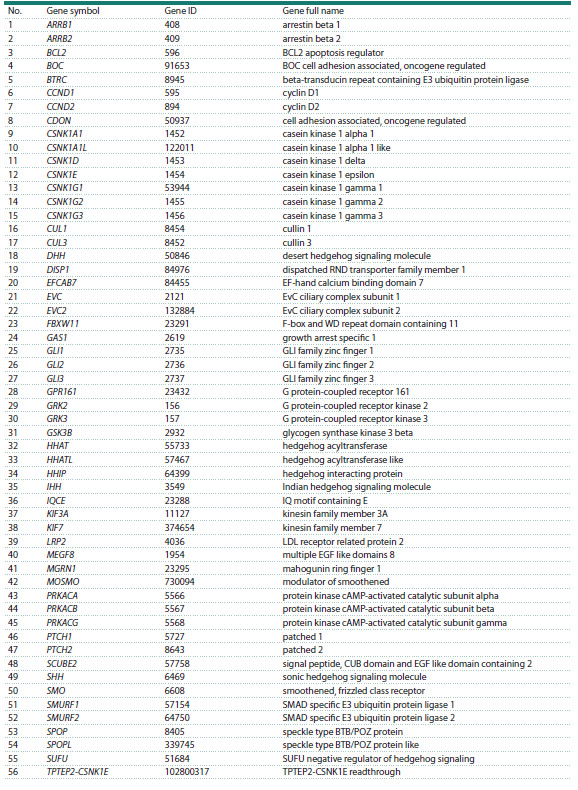
Genes of the Hedgehog signaling pathway (according to the KEGG database

To fill the “gene information” and “gene product information” blocks, data were retrieved from the NCBI Gene (https:// www.ncbi.nlm.nih.gov/gene), UniProt (https://www.uniprot. org), TRRUST (https://www.grnpedia.org/trrust/) data- bases.

Data for the “TPB affinity to the promoter” block (TBP, the TATA-binding protein, is a key regulator of transcription initiation in eukaryotic genes) was taken from the Human_ SNP_TATAdb database (Filonev et al., 2023).

The “evolutionary characteristics” block was filled using Orthoweb, a specialized software package developed to calculate two evolutionary indices: the phylostratigraphic age index (PAI) and the divergence index (DI) (Mustafin et al., 2021; Ivanov et al., 2024).

The PAI index reflects the distance of a taxon from the root of the phylogenetic tree and is calculated as the distance from the root to the node where the divergence of the species under study from the most distant related taxon occurred: the higher the PAI, the “younger” the gene in question. For human genes, PAI values range from 0 (Cellular Organisms, the root of the tree) to 15 (Homo sapiens).

The gene evolutionary variability index (DI – Divergence Index) estimates the ratio between non-synonymous sub- stitutions (which alter the encoded amino acid) in the sequences of the analyzed gene and its ortholog (dN), and synonymous substitutions (which do not change the encoded amino acid) (dS) in the nucleotide sequences of genes and their orthologs:

**Formula. 1.**
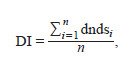
Formula 1

where dndsi is the dN/dS value for the gene and its i-th ortholog, and n is the number of orthologous genes.

The DI allows for determining the type of selection pressure acting on a given gene. DI values <1 and >1 are interpreted as evidence of stabilizing and positive selection, respectively, while DI = 1 indicates neutral evolution (Jeffares et al., 2015; Spielman, Wilke, 2015).

To construct the associative gene network and identify regulatory circuits (lower-dimensionality gene networks), the cognitive software and information system ANDSystem was used. This platform employs artificial intelligence methods to automatically extract knowledge from scientific publications and factual databases and, via the ANDVisio module, visualizes the results as a graph (Demenkov et al., 2011; Ivanisenko et al., 2015, 2019, 2022).

The gene network was reconstructed for 56 genes of the Hedgehog signaling pathway. It reflects associations with proteins encoded by these genes (“expression”), with transcription factors regulating gene expression (“expression regulation”), with proteins regulating protein transport (“transport regulation”), and with miRNAs involved in post-transcriptional regulation of protein expression (“miRNA regulation”).

Functional annotation of genes was performed using the DAVID web resource (https://davidbioinformatics.nih. gov/) (Sherman et al., 2022). This tool identifies biological processes that are statistically overrepresented in the analyzed gene set. The false discovery rate (FDR), calculated using the Benjamini-Hochberg correction, was used as the significance criterion. Only processes with an FDR < 0.05 were considered.

## Results and discussion

**The HH_Signal_pathway_db knowledge base**

**Fig. 3.**
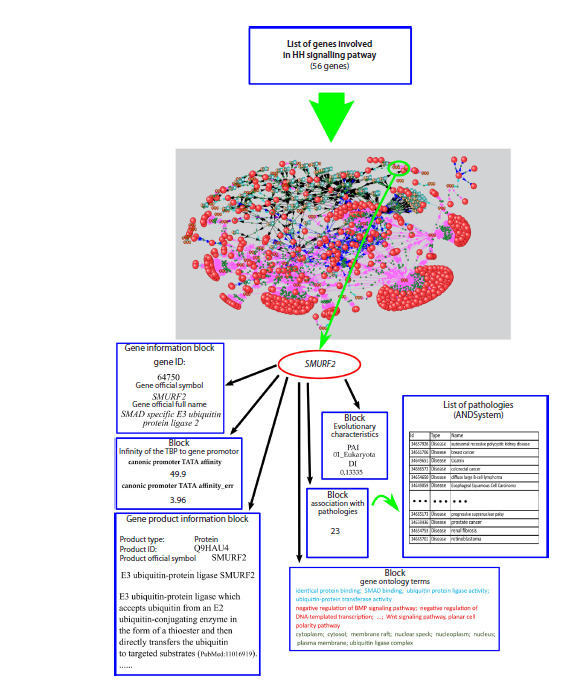
An example of filling out the HH_Signal_pathway_db knowledge base block for the SMURF2 gene.

The current version of the HH_Signal_pathway_db contains structured information on 56 human genes related to the HH pathway (Table 1). The first release of the database contains the following blocks: 1) a list of HH signaling pathway genes with links to literary sources from the PubMed database; 2) lists of proteins encoded by HH signaling pathway genes and their functions; 3) Gene Ontology terms; 4) values of gene evolutionary age indices (PAI); 5) values of gene evolutionary variability indices (DI); 6) values of TBP binding affinity to gene promoters, a key determinant of transcription intensity; 7) lists of pathologies associated with each gene; 8) a reconstructed associative gene network and the regulatory circuits identified within it. A sample of filled database blocks for a specific gene, using the SMURF2 gene as an example, is shown in Figure 3.

Below are some results of bioinformatic analysis of the information presented in the HH_Signal_pathway_db.

**Functional annotation of HH signaling pathway genes**

Analysis of biological process terms in Gene Ontology (GO) for the 56 genes performed using the DAVID resource, revealed 221 biological processes statistically significantly associated with the signaling pathway. Generally, these processes can be conditionally grouped into three main categories: morphogenesis (94), intracellular processes (60), and intercellular communication (67). Table 2. For all processes listed FDR < 0.05.

**Table 2.**
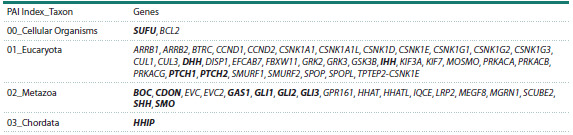
Distribution of 56 human Hedgehog signaling pathway genes according to phylostratigraphic index (PAI) values Note. Gene names belonging to regulatory circuits with feedback are highlighted in bold.

**Morphogenesis**

• GO:0042733~embryonic digit morphogenesis • GO:0042475~odontogenesis of dentin-containing tooth • GO:0007507~heart development • GO:0001658~branching involved in ureteric bud morphogenesis • GO:0003151~outflow tract morphogenesis • GO:0030324~lung development • GO:0003180~aortic valve morphogenesis • GO:0045766~positive regulation of angiogenesis • GO:0001501~skeletal system development • GO:0001942~hair follicle development • GO:0021983~pituitary gland development • GO:0001822~kidney development • GO:0001525~angiogenesis • GO:0042060~wound healing • GO:0001889~liver development • GO:0072091~regulation of stem cell proliferation etc.

**Intracellular processes**

Regulation of transcription

• GO:1902895~positive regulation of miRNA transcription • GO:1902894~negative regulation of miRNA transcription • GO:0006357~regulation of transcription by RNA polymerase II • GO:0006338~chromatin remodeling • GO:0006355~regulation of DNA-templated transcription • GO:0010468~regulation of gene expression

Response to stress

• GO:0071456~cellular response to hypoxia • GO:0034599~cellular response to oxidative stress • GO:0071466~cellular response to xenobiotic stimulus • GO:0034644~cellular response to UV • GO:0006974~DNA damage response

Regulation of cyclic processes

• GO:0048511~rhythmic process • GO:0051726~regulation of cell cycle

Apoptosis

• GO:0043066~negative regulation of apoptotic process • GO:0043065~positive regulation of apoptotic process

**Intercellular communication**

• GO:0042127~regulation of cell population proliferation • GO:0050673~epithelial cell proliferation • GO:0010595~positive regulation of endothelial cell migration • GO:0001938~positive regulation of endothelial cell proliferation • GO:0042127~regulation of cell population proliferation • GO:0072089~stem cell proliferation etc.

>Involvement in signaling pathways

• GO:0038084~vascular endothelial growth factor signaling pathway • GO:0007173~epidermal growth factor receptor signaling pathway • GO:0008543~fibroblast growth factor receptor signaling pathway • GO:0007224~smoothened signaling pathway • GO:0060070~canonical Wnt signaling pathway • GO:0030509~BMP signaling pathway • GO:0000165~MAPK cascade • GO:0007219~Notch signaling pathway • GO:0070371~ERK1 and ERK2 cascade etc.

A significant role of the Hedgehog signaling pathway is its participation in the morphogenetic processes of embryogenesis, histogenesis, and organogenesis. The pathway genes are involved in the formation of the nervous system, the development of cartilage and skeletal tissue, angiogenesis, and the development of kidneys, liver, lungs, heart, the endocrine pancreas, and genitals (Ingham, McMahon, 2001; Roy, Ingham, 2002; Fitzsimons et al., 2022; Ingham, 2022; Dilower et al., 2023).

Among the fundamental intracellular processes regulated by HH pathway genes are transcription (Gao Y. et al., 2023), response to stress stimuli (Chung et al., 2022), and maintenance of genomic stability (Ingham, McMahon, 2001). Furthermore, the signaling pathway modulates the cellular response to hypoxia, oxidative stress, and other adverse factors, which can be critical for cell survival (Kim, Lee, 2023; van der Weele et al., 2024). The involvement of Hedgehog signaling pathway elements in DNA repair (Gao Q. et al., 2019), apoptosis (Harris et al., 2011; Rimkus et al., 2016), and cell cycle regulation confirms its role in controlling cell proliferation and differentiation (Roy, Ingham, 2002).

According to available data, the HH pathway acts as a mediator of intercellular communication not only by itself; its components, in particular beta-arrestins (ARRB1/2), kinases (CCND1, CSNK1A1, CSNK1E, CSNK1A1L, GSK3B, PRKACA, PRKACB, PRKACG, TPTEP2-CSNK1E), ubiquitination proteins (BTRC, CUL1, FBXW11), and others, are involved in other signaling cascades, including MAPK/ERK, Wnt, Notch, and VEGF. The participation of HH pathway proteins in other signaling pathways has also been demonstrated by other authors (Rubin, 2007; Butí et al., 2014; Edeling et al, 2016; Luo, 2017; Fang et al., 2023).

**Associative gene network of the Hedgehog signaling pathway**

**Fig. 4.**
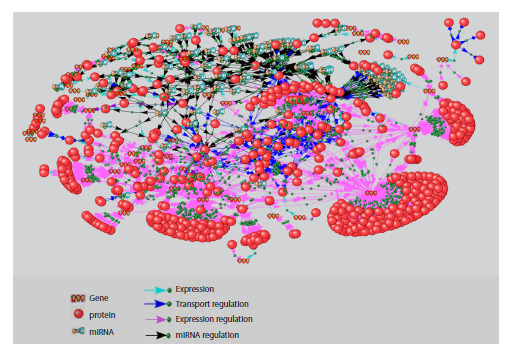
A reconstruction of the associative gene network for the human Hedgehog signaling pathway, generated by the ANDSystem tool

The network reconstructed with ANDSystem contains information on 56 genes, 504 proteins, 126 miRNAs, and 1,412 interactions of various types between its elements. A general view of the network is presented in Figure 4.

**Fig. 5.**
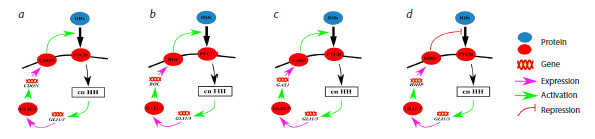
Auto-regulation of the HH signaling pathway a–c – regulatory circuits with positive feedback; d – regulatory circuit with negative feedback; SP – signaling pathway.

**Fig. 6.**
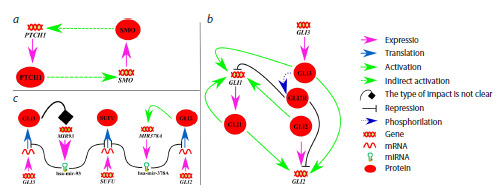
Schemes of mutual regulation of components in three regulatory circuits of the HH signaling pathway. a – regulation of PTCH1 and SMO; b – auto-regulation of GLI1/3; c – regulation of GLI2/3 and SUFU.

Analysis of the gene network revealed certain patterns pertaining to intra-network interactions. Specifically, it was shown that there are at least seven regulatory circuits within the network (Fig. 5, 6). These can be tentatively divided into two groups

The circuits of the first group mediate the auto-regulation of the signaling pathway as a whole. The second group regulates the interaction of some components within the signaling pathway itself. The first group comprises four circuits – three with positive feedback loops, implementing pathway autoactivation (Fig. 5a–c), and one with a negative feedback loop, mediating autorepression of the pathway (Fig. 5d). The auto-activation circuits include the membrane proteins GAS1, BOC, CDON, which participate in the interaction of the PTCH1/2 receptor with its HH ligand, thereby facilitating signal transduction. The expression of the genes encoding these membrane proteins is controlled by the GLI1/3 transcription factors (Allen et al., 2007; Song et al., 2015; Echevarría- Andino et al., 2023).

The main component of the fourth circuit is the HHIP protein, which prevents the binding of PTCH1/2 to HH, thereby prohibiting signal propagation. The HHIP gene is a target of GLI1/3 transcription factors (Chuang, McMahon, 1999; Falkenstein, Vokes, 2014).

The second group, defining the character of certain interactions within the HH pathway, is formed by three circuits. The first controls the interaction between PTCH1 and SMO via a positive feedback loop (Fig. 6a). The second is a mutual regulation circuit of the genes encoding the GLI1/3 transcription factors (Fig. 6b). It can exist in two states depending on the functional status of the pathway. In the presence of the HH signal, the circuit operates in a mode of mutual gene activation via positive feedback loops. In the absence of the signal, the repressor form GLI3R suppresses the transcription of the GLI1/2 genes and turns off the auto-activation. Thus, the balance between the activator and repressor forms of GLI is maintained (Wang et al., 2000; Vokes et al., 2007; Briscoe, Thérond, 2013). The third circuit of the group functions with the participation of two miRNAs – hsa-mir-93 and hsa-mir-378A, regulating the levels of GLI2/3 and SUFU via negative feedback loops (Fig. 6c). The involvement of miRNAs, including hsa-mir-93 and hsa-mir-378A, in regulating the expression of HH pathway proteins was established by A. Helwak et al. (2013). Analysis of the reconstructed HH signaling pathway gene network revealed that the genes encoding these miRNAs are targets for the GLI2/3 transcription factors

**Evolutionary characteristics of human Hedgehog signaling pathway genes:**

**Fig. 7.**
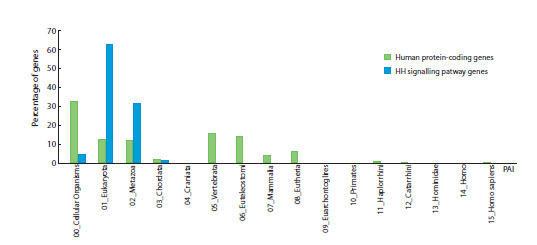
Distribution of PAI values among genes of the Hedgehog signaling pathway (56 genes) and all human protein-coding genes (19,491 genes). The differences in values are statistically significant at p < 0.05 according to the Mann–Whitney test.

The distribution of genes by values of their phylostratigraphic indecies PAI is presented in Table 2 and Figure 7.

The vast majority of pathway genes are characterized by indices of PAI = 01 (35 genes) and PAI = 02 (18 genes), indicating their emergence at the level of the first unicellular eukaryotes and the first multicellular animals. Two genes – BCL2 and SUFU – originated significantly earlier – at the cellular level of biological organization (their PAI = 00). Both of these genes control the cell pool – BCL2 as an apoptosis regulator, and SUFU as an inhibitor of tumor growth, i. e., uncontrolled cell proliferation (Willis et al., 2003; Cheng, Yue, 2008).

Only one gene, HHIP, originated during the formation of chordates, has a PAI value of 03. The eponymous protein inhibits the signaling cascade already at its initial stage by binding to the PTCH1 receptor and preventing the ligand– receptor interaction.

Previously, independent data on the emergence time of certain components of the human Hedgehog (HH) signaling pathway prior to vertebrate divergence had been obtained for all HH ligands (Kumar et al., 1996) and for the GLI transcription factors (Shimeld et al., 2007), and these findings are consistent with the results presented.

A comparison of the PAI value distribution between HH cascade genes and all human protein-coding genes (Fig. 7) showed a statistically significant bias towards more ancient values in HH pathway genes (p < 0.05, Mann–Whitney test). This aligns with the fact that this pathway is activated earlier than others in ontogeny, suggesting that its core components therefore had to emerged at early stages of multicellular organisms evolution. Indeed, all forms of HH, GLI, PTCH, and SMO proteins, which play the main role in signal transduction, are characterized by PAI = 01–02, and their functional analogs are present even in invertebrate animals (Ingham, McMahon, 2001; Wilson, Chuang, 2010). Notably, all genes of the regulatory circuits except HHIP, have ancient origin, at that HHIP is the only gene included in the regulatory circuit with negative feedback.

**Fig. 8.**
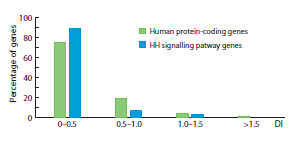
Distribution of DI values for Hedgehog signaling pathway genes compared to all human protein-coding genes.

Figure 8 shows the distribution of DI index values for HH pathway genes. Given that this pathway orchestrates the implementation of fundamental cellular processes involved in morphogenesis, including division, differentiation, and apoptosis, it is unsurprising that 89 % of its genes (50) have a DI index <0.5, with 12 of them (≈21 %) having an index below 0.1. This fact confirms that the signaling pathway, and the genes of the regulatory circuits governing its function, are under stabilizing selection which limits the accumulation of genomic changes.

In the analyzed set of 56 genes, only two have DI > 1 – these are CSNK1A1L (1.213) and EFCAB7 (1.051). This finding, within the framework of the applied method, suggests that these genes may be under positive selection. The kinase CSNK1A1L phosphorylates GLI1/3 proteins. According to KEGG database data (hsa04340), in the human HH signaling pathway, several other kinases (CSNK1A1, CSNK1D, CSNK1E, CSNK1G1, CSNK1G2, CSNK1G3, TPTEP2-CSNK1E), encoded by genes of the same name, also participate in this process. All of them fall into the group with PAI = 02_Eukaryota, however, the DI values for them range from 0.0361 for CSNK1A1 to 0.264 for CSNK1D, indicating the action of stabilizing selection on them. It can be assumed that CSNK1A1L might have “incorporated” into the signaling pathway later in evolution than the other kinase genes, and therefore may currently be experiencing the influence of positive, rather than stabilizing, selection.

The EFCAB7 protein, together with EVC, EVC2, and IQCE proteins, is involved in anchoring SMO to the primary cilium of mammalian cells, which distinguishes the signal transduction mechanism from the analogous process in Drosophila, whose cells do not possess primary cilia (Chen et al., 2009; Gorojankina, 2017). Probably, the weak pressure of positive selection on the EFCAB7 gene, reflected in its DI value close to one, is related precisely to the later emergence of the mechanism involving primary cilia in the signal transduction process compared to other pathway components performing the same function – the EVC, EVC2, and IQCE genes (Chen et al., 2009; Wilson, Chuang 2010), which are evidently under stabilizing selection, as indicated by their DI values of 0.298, 0.421, and 0.679, respectively

Thus, the overwhelming majority of Hedgehog signaling pathway genes can be characterized as ancient, subject to stabilizing selection, preventing the accumulation of genetic variability and promoting functional stability of the genes. Their conservatism confirms the critical role of the HH pathway in regulating fundamental ontogenetic processes.

## Conclusion

A prototype of the HH_Signal_pathway_db knowledge base has been developed. It accumulates information on the structural and functional organization of the evolutionarily conserved Hedgehog (HH) signaling pathway in humans, integrating data from KEGG, NCBI Gene, UniProt, and other sources. The database systematizes fragmented data on the HH signaling pathway in humans and can serve as a tool for systematic analysis of its role in ontogenesis, maintaining homeostasis, and pathology development.

The bioinformatic analysis of some data from the base, in particular, showed that: 1) according to functional annotation, the pathway’s genes are associated with three categories of processes: intracellular, organ morphogenesis, and intercellular communication, including interaction with other signaling cascades; 2) the vast majority of the pathway’s genes are of ancient origin and subject to stabilizing selection; 3) the reconstructed associative gene network of the HH pathway contains 56 genes, 504 proteins, 126 miRNAs, and establishes 1,412 interactions among them; 4) the network’s functioning is regulated by seven regulatory circuits – five with positive and two with negative feedback. One of the negative feedback circuits involve two miRNAs.

## Conflict of interest

The authors declare no conflict of interest.
